# Next generation methods and tools for timing the evolution of species and strains

**DOI:** 10.1186/1471-2164-15-S2-O13

**Published:** 2014-04-02

**Authors:** Sudhir Kumar, Koichiro Tamura

**Affiliations:** 1 Center for Evolutionary Medicine and Informatics, Biodesign Institute, Arizona State University, Tempe, AZ 85287-5301, USA; 2 School of Life Sciences, Arizona State University, Tempe, AZ 85287-4501, USA; 3 Research Center for Genomics and Bioinformatics, Tokyo Metropolitan University, Tokyo 192-0397, Japan; 4 Center of Excellence in Genomic Medicine Research, King Abdulaziz University, P.O. Box: 80216 Jeddah 21589, Kingdom of Saudi Arabia; 5 Department of Biological Sciences, Tokyo Metropolitan University, Tokyo 192-0397, Japan

## 

Molecular dating of species divergences has become an important means to add a temporal dimension to the Tree of Life. Increasingly larger datasets encompassing greater taxonomic diversity are becoming available to generate molecular timetrees by using sophisticated methods that model rate variation among lineages. However, the practical applications of these methods are challenging because of the exorbitant calculation times required by current methods for contemporary data sizes, the difficulty in correctly modeling the rate heterogeneity in highly diverse taxonomic groups, and the lack of reliable clock calibrations and their uncertainty distributions for most groups of species. I will present our published RelTime method that estimates relative times of divergences for all branching points (nodes) in very large phylogenetic trees without assuming a specific model for lineage rate variation or specifying any clock calibrations. RelTime performed better than existing methods when applied to very large computer simulated datasets where evolutionary rates were varied extensively among lineages by following auto-correlated and uncorrelated rate models. I will also discuss some preliminary results aimed at assessing the prospects for building large species time trees when the gene sequence coverage is incomplete. Our results indicate that the estimates of divergence times are surprisingly good even when data from a majority of genes are missing for many species, as long as multiple genes are available from each species. The speed and accuracy of RelTime will enable molecular dating analysis of very large datasets, which will be useful for determining the relative ordering and spacing of speciation events, identifying lineages with significantly slower or faster evolutionary rates, diagnosing the effect of selected calibrations on absolute divergence times, and estimating absolute times of divergence when highly reliable calibration points are available.

